# A Systematic Review and Meta-Analysis of Rituximab in Antibody-mediated Renal Allograft Rejection

**Published:** 2011-05-01

**Authors:** G. Hychko, A. Mirhosseini, A. Parhizgar, N. Ghahramani

**Affiliations:** 1 *Indiana University, *; 2 *Pennsylvania State University College of Medicine, USA *

**Keywords:** Antibody-mediated rejection, Rituximab, CD20, Kidney, transplantation, Meta-analysis, Systematic review

## Abstract

Background: The standard treatment of antibody-mediated rejection (AMR) consists of antilymphocyte antibody, intravenous immunoglobulin, and plasmapheresis. This treatment is associated with a high rate of resistance and refractory AMR. Recent interest has focused on use of rituximab (RTX), a chimeric anti-CD20 monoclonal antibody.

Objective: We conducted a systematic review and meta-analysis of studies of RTX in AMR of the renal allograft.

Methods: Combining two comprehensive search themes (AMR and RTX), we searched electronic databases from 1969 through 2010, supplemented by a manual review of abstracts from nephrology and transplant meetings, and reference lists of review articles. All studies evaluating explicit response of patients with AMR to RTX were included. The outcome was pooled odds ratio (OR) of response to RTX.

Results: A total of 114 studies were identified, 94 of which were excluded on initial screening. Analysis of the 10 studies (249 patients) showed an OR of 3.16 (95% CI: 1.75–5.70) for response to RTX. Reported adverse effects included BK virus nephropathy, cytomegalovirus (CMV) viremia, pneumonia, herpes zoster, and septic shock.

Conclusion: This study suggests that RTX is a reasonable therapeutic option in the treatment of AMR. Further randomized studies are necessary to establish its efficacy and safety.

## INTRODUCTION

Antibody-mediated rejection (AMR) is defined by four criteria: morphological evidence of acute tissue injury; immunopathological evidence of antibody mediation; C4d staining in peritubular capillaries; and serological evidence of circulating donor-specific antibodies (DSA) [1]. The reported incidence is 8%–10% and among all episodes of acute rejection, up to 1/3 show features of AMR [2]. AMR has significant effect on long-term graft survival [3-8] and it is associated with a greater frequency of allograft dysfunction and graft loss [9]. Treatment of AMR remains a challenge. Traditional anti-rejection treatments, such as administration of high dose steroids and anti-lymphocyte antibodies are usually ineffective, since they are primarily directed toward cellular immune mechanisms. More recently, combination of anti-lymphocyte antibody, intravenous immunoglobulin (IVIG), and plasmapheresis have been used [10]. 

Rituximab (RTX) is a chimeric murine/human anti-CD20 antibody [11]. It binds human CD20 with high affinity [12] and directly inhibits B-cell proliferation by antibody-dependent, cell-mediated, and complement-mediated cytotoxicity [13-15]. Findings from several studies suggest that the use of RTX, alone or in combination with IVIG, plasmapheresis and steroids may improve outcomes in severe, steroid-resistant or antibody-mediated rejection episodes [16-27].

The majority of studies comparing treatment of AMR have been either retrospective or non-randomized trials with small sample sizes. To the best of our knowledge, no systematic reviews or meta-analyses have addressed use of RTX in treatment of AMR. In this study, we conducted a systematic review and meta-analysis of all studies to examine the effect of RTX on treatment of AMR.

## METHODS

Literature search:

We developed two comprehensive search themes and combined them using the Boolean operator “AND.” The first theme, AMR, was created by using the following terms: “antibody mediated rejection” or “humoral rejection.” The second theme, RTX, was created by using the words: “Rituximab” or “anti CD20.” Using this search strategy in electronic databases (Medline, ISI Web of Science, Cochrane Central Register and Dissertation Abstracts) from 1969 through 2010, we sought to identify prospective or retrospective studies evaluating the safety and efficacy of rituximab in AMR. References of identified articles were scanned to identify other possible studies. The search was also supplemented by a search of abstracts from the proceedings of the American Society of Nephrology and American Transplant Congress.

Inclusion and exclusion criteria:

All clinical studies evaluating the effect of RTX on AMR with explicit reporting of the response were included irrespective of the number of patients, journal type or language of publication. Exclusion criteria consisted of: 1) studies with insufficient data; 2) duplicate studies; 3) single case reports; and 4) review articles. 

Study selection and data extraction:

Two of the authors (GH and NG) independently evaluated articles for eligibility in a two-stage procedure. In the first stage, the abstracts of all identified studies were reviewed. The second stage consisted of full-text review of studies that met the inclusion criteria or those for which eligibility was uncertain. Articles that were selected by either individual were reviewed by both authors and evaluated on both inclusion and exclusion criteria. Disagreement between authors was resolved by consensus. Data extracted included study design, details of treatment, response to treatment and adverse effects.

Study Outcomes:

The primary outcome was the pooled estimate of the odds ratio (OR) of response to RTX. Response was defined by at least partial improvement in the graft function. Adverse effects were reviewed and noted but not compared due to variations in reporting.

Meta-analysis:

We used the Mantel-Haenszel model to estimate the pooled OR with 95% confidence intervals (CI) for study outcomes under the fixed effect model, using data from all eligible studies. The presence of heterogeneity across trials was evaluated using Q-statistic for heterogeneity. The heterogeneity statistic was then incorporated to calculate the summary OR under the random effects model. All statistical analyses were performed using Comprehensive Meta-Analsyis ver 2.2.050 (Engelwood, NJ).

## RESULTS

**Figure 1:**
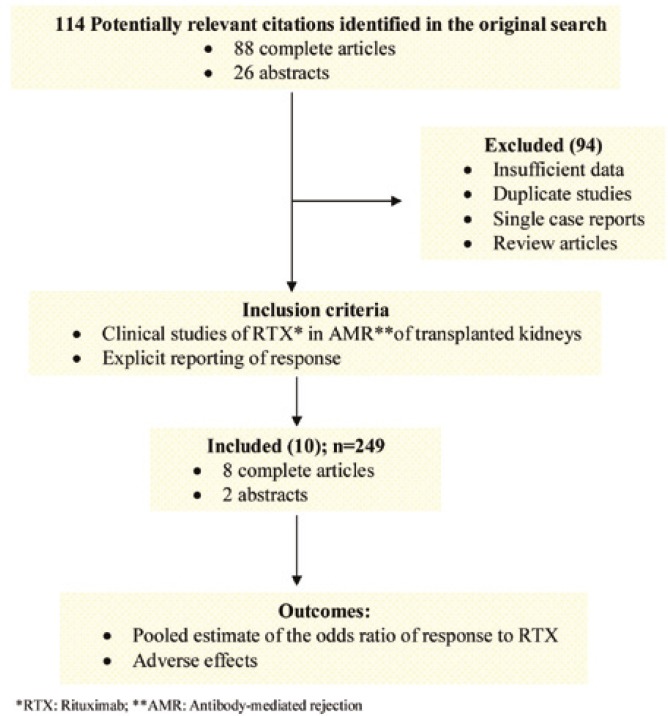
Identification and selection of studies

**Figure 2:**
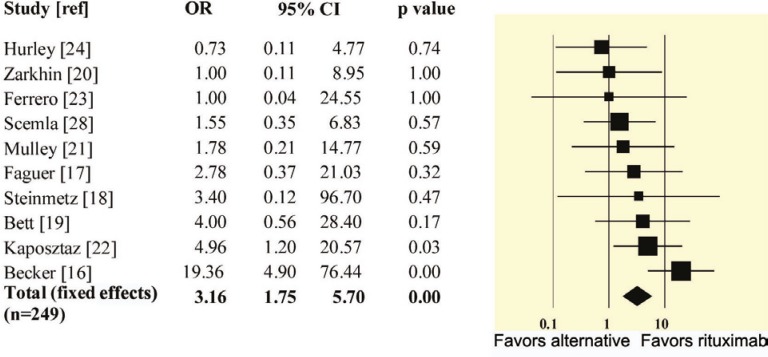
Odds retio of response to rituximab *vs *alternative treatment

Figure 1 summarizes the process used to identify and select the studies for the systematic review. The initial search yielded 114 potentially relevant citations, of which 94 were excluded for reasons shown. There was complete agreement between the authors for the studies that were selected for review and meta-analysis. Tables 1 and 2 illustrate the summary and individual characteristics of the included studies, respectively. The 10 included studies (two abstracts and eight complete articles) consisted of six prospective and four retrospective studies and included a total of 249 patients. One of the complete articles with the primary outcome of adverse infectious effects [28] was deemed appropriate for inclusion. It had explicit comparative reporting of graft outcome between patients with AMR receiving RTX and alternative treatment. Figure 2 shows the OR of response for each study, as well as the pooled estimate of the OR using the fixed effects model (Q = 12.23, p=0.2; random effects: 2.91 (95% CI: 1.43–5.91)). Six of the studies explicitly reported on adverse infectious effects (Table 3) [17, 18, 20-22, 28]. Three of these studies which compared RTX with other treatments did not find an increased incidence of infectious complications compared with alternative treatment [20, 22, 28]. The other three studies reported seven types of serious adverse effects among 11 patients. The adverse effects included BK virus associated nephropathy (BKVAN) (n=5), septic shock (n=2), pyelonephritis (n=1), PD peritonitis (n=1), pneumonia (n=1), herpes zoster (n=1) and CMV viremia (n=1). One of the patients was reported to have concomitant CMV viremia and BK virus associated nephropathy [21].

**Table 1:** Summary characteristics of identified studies

**Type of study**	**Complete articles**	**Abstracts**	**Total**	**No. of Patients**
All studies	8	2	10	249
Prospective	5	1	6	106
Retrospective	3	1	4	143
*De novo* use of RTX	6	1	7	179
RTX used for refractory AMR	2	1	3	70
Pediatric	1	0	1	20
Adult	7	2	9	229

RTX: Rituximab

AMR: Antibody-mediated rejection

**Table 2:** Characteristics of individual studies

**Study**	**Publication Year**	**Design**	**Study Outcome(s)**	***n***	**Follow-up (months)**
Becker [16]	2004	Prospective*	graft survival	27	24
Faguer [17]	2007	Prospective	B cell depletion; graft function	8	10
Steinmetz [18]	2007	Retrospective; comparative	B cell depletion; creatinine; Bx**	16	3
Bett [19]	2008	Retrospective	Creatinine	9	46
Zarkhin [20]	2008	Prospective; comparative; pediatric	B cell depletion; graft survival; Bx; DSA@	20	12
Mulley [21]	2009	Prospective*	B cell depletion; creatinine	7	21
Kaposztaz [22]	2009	Retrospective; comparative	graft survival; graft function; Bx; creatinine	54	24
Ferrero [23]	2010	Prospective; comparative	Creatinine	8	10
Hurley [24]	2010	Prospective*	graft survival; creatinine	36	24
Scemla [28]	2010	Retrospective	graft survival	64	25

* Rituximab used in refractory antibody-mediated rejection

** Bx: biopsy

@ DSA: Donor specific antibody

**Table 3:** Adverse infectious effects

**Study**	**Number of patients**	**Adverse infectious effects**
Mulley [21]	7	CMV* viremia + BKVAN** (n=1), pneumonia (n=1), Herpes zoster (n=1),
Faguer [17]	8	BKVAN (n=3), septic shock (n=2), pyelonephritis (n=1), PD† peritonitis (n=1)
Steinmetz [18]	16	BKVAN (n=1)
Zarkhin [20]	20	No difference in incidence *vs.* non-RTX‡ group
Kaposztaz [22]	54	No difference in incidence *vs.* non-RTX group
Scemla [28]	64	No difference in incidence *vs.* non-RTX group

* CMV: Cytomegalovirus;

** BKVAN: BK virus-associated nephropathy;

† PD: Peritoneal dialysis;

‡ RTX: Rituximab

## DISCUSSION

This meta-analysis of 10 studies, including 249 patients, suggests that treatment with RTX is associated with improved graft outcome in patients with AMR of the transplanted kidney. It is noteworthy that the favorable effect of RTX is driven by the two relatively larger studies. One of these studies [16] evaluated the effect of RTX in refractory AMR and the other one [22] was a study comparing “RTX + plasmapheresis” with “plasmapheresis alone”. Six of the studies specifically reported on adverse effects. Among these, the three larger studies [20, 22, 28] with a total of 138 patients, reported the incidence of infectious complications to be similar to standard treatment or controls. The most commonly reported serious infectious adverse effect was BKVAN. While none of the studies included in this meta-analysis have mentioned *Pneumocystis jiroveci* infection, three cases of *Pneumocystis* pneumonia have been reported following use of RTX for AMR [29, 30], as well as among patients receiving RTX for treatment of lymphoma [31-36]. These findings have prompted the suggestion that *Pneumocystis jiroveci* prophylaxis be considered in patients receiving RTX [30]. While the main focus of adverse reactions has been infectious complications, another serious reported side effect of RTX is interstitial pneumonitis [37-39] with no clear cause and effect association. In transplanted patients receiving multiple complex medications it is difficult to ascertain the adverse effects of a single agent, such as RTX. This is particularly challenging in the case of adverse infectious complications, since transplanted patients have received multiple other agents that could have potentiated a severe infectious process. Overall, our review of larger studies that have explicitly attempted to ascertain differences in adverse effects does not support a higher incidence of adverse effects with RTX use. However, as is true with use of all immunosuppressant medications, close observation of patients on RTX for adverse events is necessary. The limitations of our meta-analysis include: 1) the paucity of randomized trials and prospective studies; 2) lack of uniformity in the definitions of response among studies; 3) heterogeneity in the “standard treatment” among studies; and 4) the inherent limitation of any meta-analysis in the ability to perform multivariate analyses. 

We conclude that while RTX is an appealing option in the treatment of AMR, it is important to recognize the knowledge gaps in efficacy and safety of its use. This systematic review underscores the need for well-designed, long-term and adequately powered prospective studies which would potentially elucidate various potential confounders and would address not only early response, but also long-term graft and patient survival. 
